# A checklist of vascular plants and uses of some species for livelihood-making in Setiu Wetlands, Terengganu, Malaysia

**DOI:** 10.3897/phytokeys.160.52946

**Published:** 2020-09-08

**Authors:** Jamilah Mohd Salim, Gaik Ee Lee, Muhamad Razali Salam, Salwa Shahimi, Elizabeth Pesiu, Jarina Mohd Jani, Nurul Amira Izzaty Horsali, Rohani Shahrudin, Siti Mariam Muhammad Nor, Ju Lian Chong, Faridah Mohamad, Akmal Raffi, Dome Nikong

**Affiliations:** 1 Institute of Tropical Biodiversity and Sustainable Development (Bio-D Tropika), Universiti Malaysia Terengganu, 21030 Kuala Nerus, Terengganu, Malaysia Universiti Malaysia Terengganu Terengganu Malaysia; 2 Faculty of Science and Marine Environment, Universiti Malaysia Terengganu, 21030 Kuala Nerus, Terengganu, Malaysia Universiti Malaysia Sarawak Kuching Malaysia; 3 Mangrove Research Unit, Institute of Oceanography and Environment, Universiti Malaysia Terengganu, 21030 Kuala Nerus, Terengganu, Malaysia Universiti Malaysia Terengganu Terengganu Malaysia; 4 Faculty of Resource Science and Technology, Universiti Malaysia Sarawak, 94300 Kota Samarahan, Sarawak, Malaysia Universiti Malaysia Sarawak Kuching Malaysia

**Keywords:** coastal ecosystem, diversity, flora, local community, Malesia, useful plants

## Abstract

The Setiu Wetlands, a unique area with nine interconnected habitats, comprises a considerable fraction of the total Peninsular Malaysia’s wetland flora. Although botanical collecting in the area has been active in the past 10 years, only a few studies dealing with the wetland flora have been published. Thus, a detailed checklist of this area is urgently needed to ensure the continuity of its inter-relating flora and fauna, as well as the livelihood of the local people. In this work we conducted a survey of the vascular plant flora of Setiu Wetlands and investigated the most important plants used by the local communities. Our checklist accounts for 406 taxa from 277 genera and 106 families, including 24 (6%) species of ferns and lycophytes, three gymnosperms, 257 (64%) dicotyledons and 122 (30%) monocotyledons. This comprehensive plant checklist will be a primary reference for the management of the newly gazetted Setiu Wetlands State Park covering more than 400 hectares of lands and water bodies.

## Introduction

**Figure 1.**
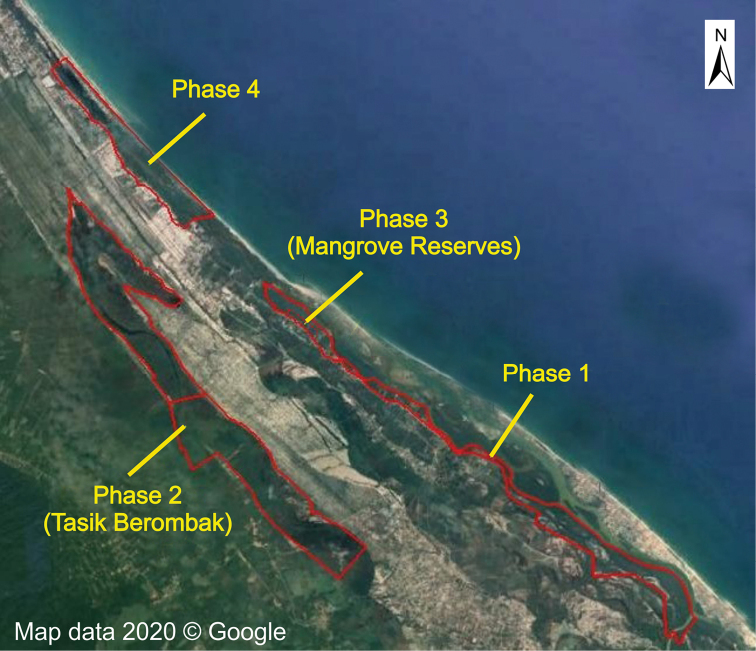
The boundaries (red line) of the forest to be gazetted in Setiu Wetlands as state park. Map courtesy of the Terengganu State Parks.

Wetlands are not only among the most productive and complex ecosystems (Costanza et al. 1997), but are also known to benefit humans with significant economic and ecological values (Barbier et al. 2011). The importance of wetlands has increased tremendously following the 2004 catastrophic tsunami which affected many places severely in the Asian region. The Setiu Wetlands (SW) constitutes the largest wetland complex in the east coast of Peninsular Malaysia which is located in an arbitrary but exclusive zone referring to the larger Setiu district in Terengganu. The coastal lagoon is the largest part of the SW, stretching approximately 14 km, parallel to the coastline, from Lembah Bidong in the south up to Beting Lintang to the north, while the wetland basin covers about 23,000 ha of lands and 880 ha of water bodies (Nakisah and Fauziah 2003). In 2018, in lieu of protecting vital catchment areas and their natural heritage, the state government of Terengganu gazetted two new state parks, one of which was in the Setiu district. Driven by its importance for the local economy and the dire need to wisely manage SW for the sustainability, efforts to legally protect SW were initiated more than 20 years ago. However, it was not until recently that the state authority of Terengganu passed the Terengganu State Park Enactment 2017, under which, 432 ha of SW were gazetted as State Park in Phase 1 covering mainly the SW brackish lagoon and estuary (Fig. 1). In the near future, the gazettement for three more phases of this State Park will cover possibly one of the largest coastal freshwater lakes in Peninsular Malaysia, locally known as Tasik Berombak. Tasik Berombak is hydrologically important by supplying the primary source of freshwater into the brackish lagoon of SW (Sathiamurthy 2015) which is a hub for economic and livelihood activities of the SW local community. In addition, phases 3 and 4 of the gazettement intend to cover mostly mangrove islands in SW, but many issues and challenges, primarily related to land title, need to be addressed.

**Figure 2.**
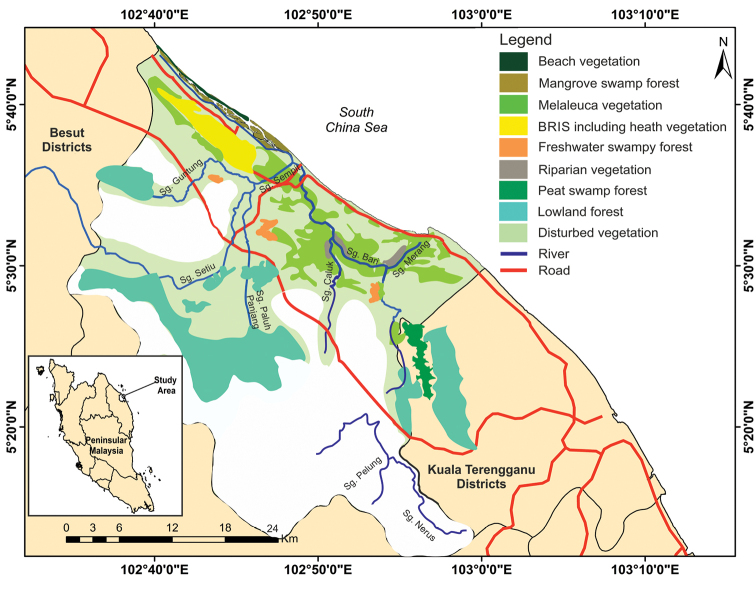
The locality of Setiu Wetlands and the nine vegetation types.

SW supports major wetlands ecosystem of marine, coastal vegetation, brackish and freshwater swamps with nine interconnected habitats of sea, beach, mudflats, lagoons, estuaries, rivers, islands, coastal and mangrove vegetation (Mohd Lokman and Sulong 2001; Nakisah and Fauziah 2003; Jamilah et al. 2014). The nine vegetation types (Fig. 2) including the beach-ridge vegetation or BRIS soil vegetation are lowland forest, mangrove swamp forest, peat swamp forest, freshwater swamp forest, riparian vegetation, beach vegetation, heath vegetation (coastal dunes forest), and disturbed vegetation. Each of the habitats is characterised by a unique yet intricate physical environment, supporting its biological entities. Intimate and complex interaction between wetlands, people and the environment could clearly be observed in Setiu district where most of the natural resources harvested from SW are vital for supporting local livelihoods (Faridah et al. 2015). Similar to other wetlands, SW integrity critically depends on the physical and biological environments. Vegetation or flora are the vital biological entity of the SW with many efforts conducted to document this entity (for example, Jamilah et al. 2014; Siti Fatimah et al. 2015; Razali et al. 2017; Rohani et al. 2017). Furthermore, the SW flora is edaphically adapted, for example, the BRIS soil vegetation which is largely confined to the sandy environment of Terengganu narrow coastal stripe and such unique vegetation is not found on the west coast of Peninsular Malaysia (Jamilah et al. 2014).

Setiu lagoon supports several islets within the lagoon with healthy mangrove vegetation. However, the mangrove ecosystem here is also characterised by a relatively sandier habitat as compared to the typical mangroves on the west coast of Peninsular Malaysia such as the Matang mangroves. Setiu mangroves are not only supporting the three classes of common mangrove vegetation, namely exclusive, non- exclusive and associate (Japar 1994), but also additional vegetation associated with sand ridges. This vegetation, including vascular plants, offers significant ecosystem services, such as providing food and shelter to animals residing in the area, with many being economically important. Furthermore, Setiu lagoon, which is the longest brackish wetlands in Terengganu, includes several patches of sandy-mudflats which support a healthy community of bivalves [including *Scapharca cornea* (local name: kepah bulu) and *Meretrix meretrix* (kepah minyak)] (Wan Bayani and Zaleha 2015) that are harvested by the Setiu locals for sale (Faridah et al. 2016). Two species of seagrasses (*Halodule pinifolia* and *Halophila minor*) are recorded to thrive well here (Syarifah et al. 2008). This seagrass-mangrove continuum is reported to be an important nursery ground for the juveniles of fishes such as grouper, and pink ear emperor fish, *Lethrinus lentjan* (Le et al. 2018). The mangroves of Setiu, and its lagoon, are also an important habitat for highly demanded mangrove crabs, *Scylla* spp. (Ikhwanuddin et al. 2010), and is the source of income for many local fishermen in the area. The geography and the vegetation cover of the area support it as a hub for aquaculture activities, covering brackish water cage culture, pond culture, pen culture especially on groupers, and also oyster farming (Suratman et al. 2014).

In terms of soil origin, the Setiu coastal plain originated from marine-based deposit, arranged in a series of ridges and depressions parallel to the shoreline (Ali and Mohamed 2007; Sathiamurthy 2015) known as BRIS (Beach Ridges Interspersed with Swales). BRIS is oligotrophic or infertile and unsuitable for agriculture (Lim 2002) partly due to extreme water retention capacity and comprises 90% sand (Mohd Ekhwan et al. 2009). However, BRIS soil supports a distinct natural vegetation formation which is different from a typical evergreen rainforest (Jamilah et al. 2013). The ridge part supports heath-like ecosystem plants, while the depression site is usually a pocket of seasonal wetland with adapted vegetation (Jamilah et al. 2011).

The nine habitats in SW are increasingly being exposed to various anthropogenic and natural pressures. This could threaten the integrity and ability of those natural ecosystems to fulfil their ecological roles for the benefit of the local community and the coastal environment. As vegetation is the most important entity of the wetlands that supports other important life forms, it is essential to highlight the diversity of flora in SW. The aims of this paper are to provide the first comprehensive checklist of vascular plants of SW, and to understand the use of wild plants for livelihood continuity and sustainability in SW. The latter also further aims to understand how the local community’s utilisation affects the plants’ sustainability, so that sustainable resource management and conservation policy for SW can be achieved.

## Materials and methods

The checklist is based on the plant collections carried out by JMS, EP, SMMN and DN with the help of field assistant, MRS. More than 30 different localities were visited after 2010 in the nine different ecosystems of SW. Specimens were deposited at the Herbarium of Universiti Malaysia Terengganu (UMTP). In addition, the checklist is also based on a search of the literature (e.g., Mohd Lokman and Sulong 2001; Jamilah et al. 2014; Siti Fatimah et al. 2015; Razali et al. 2017; Rohani et al. 2017; Pesiu 2018) as well as herbaria that store collections of specimens collected from SW, such as the Forest Research Institute of Malaysia, Kepong (KEP) and the Herbarium of Universiti Kebangsaan Malaysia, Bang (UKMB). The checklist includes family, species and local names, and life forms. It also provides the conservation status according to the IUCN Red List of Threatened species (IUCN 2020), Malaysia Plant Red List, Peninsular Malaysia Dipterocarpaceae (Chua et al. 2010), Malaysia Biodiversity Information System (MyBIS) and Convention on International Trade on Endangered Species of Wild Fauna and Flora (CITES).

A total of 188 houses from six villages, i.e. Beting Lintang, Gong Batu, Pengkalan Gelap, Fikri, Mangkok and Penarek, were opportunistically selected for a rapid livelihood survey to determine their dependence on SW wild flora resources. In addition to that, a stratified sampling of 10 households belonging to identified resource users was later conducted in Beris Tok Ku, to provide a better representation of wild flora resource utilisation in the area.

## Results and discussion

### Families, genera and species diversity

**Figure 3.**
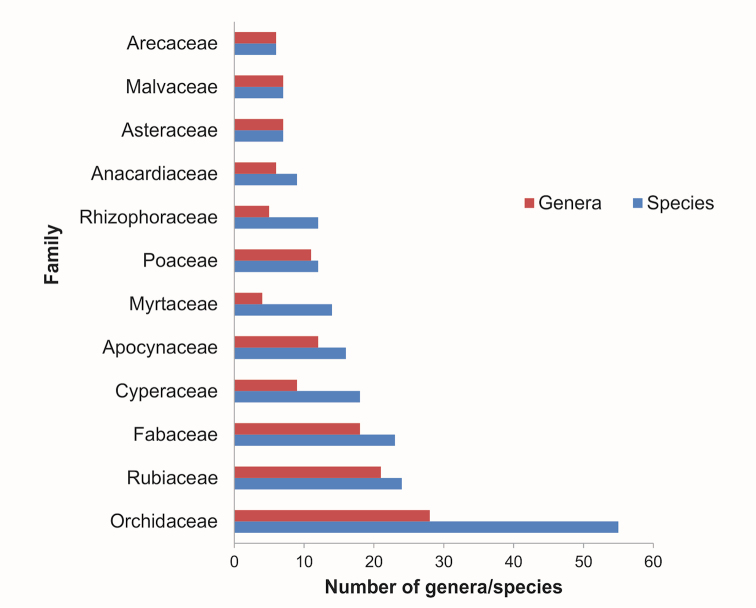
The 12 largest families and genera of the vascular plants of Setiu Wetlands.

We recorded 406 taxa (400 species, three varieties and three hybrids) from 277 genera and 106 families of vascular plants in the nine habitats of SW, including 24 species of ferns and lycophytes, three species of gymnosperms (*Cycas edentata*, *Gnetum cuspidatum* and *G. gnemon*), with 257 being dicotyledons, and 122 monocotyledons (Table 1). This represents 19% of 2168 species recorded growing in wetlands of Peninsular Malaysia (Said and Zakaria 1992) and also illustrates the fact that SW flora is relatively species rich. The most speciose family recorded from SW is Orchidaceae (56 species/28 genera), followed by Rubiaceae (24 species/20 genera) and Fabaceae (22 species/17 genera) (Fig. 3), while there are 43 families represented only by a single species e.g., Amaryllidaceae, Commelinaceae, Cycadaceae, Dioscoreaceae, Flagellariaceae and Pittosporaceae (see Appendix 1 for other families). Among the genera that contribute most to the total number of species are *Dendrobium* (11 species), *Bulbophyllum* and *Syzygium* with 10 species, while *Bruguiera*, *Cyperus* and *Sonneratia* have five species each. In terms of the life forms (Table 2), trees have the highest percentage (39.7%) followed by terrestrial herbs and epiphytes with 16.5% and 13.3% of the taxa, respectively. Apart from the trees, the herbaceous species which can be terrestrial, epiphytic or climbing, are represented by 27.8% of the species, which implies that trees and herbaceous flora are the most important components of the SW areas.

**Table 1.** Number of families, genera and species from Setiu Wetlands, Terengganu.

	Families	Genera	Species
Ferns and lycophytes	12	16	24
Gymnosperms	2	2	3
Dicotyledons	73	191	257
Monocotyledons	19	70	122
Total	106	277	406

**Table 2.** Number of species from Setiu Wetlands according to their life form.

Life form	No. of species	Percentage (%)
Trees	161	39.7
Terrestrial herbs	67	16.5
Epiphytic herbs and shrubs	54	13.3
Shrubs	39	9.6
Climbing herbs and shrubs	33	8.1
Ferns	23	5.7
Aquatic herbs	15	3.7
Palms	5	1.2
Parasitic herbs and shrubs	5	1.2
Palm-like (*Pandanus* spp.)	4	1
Total	406	100

**Figure 4.**
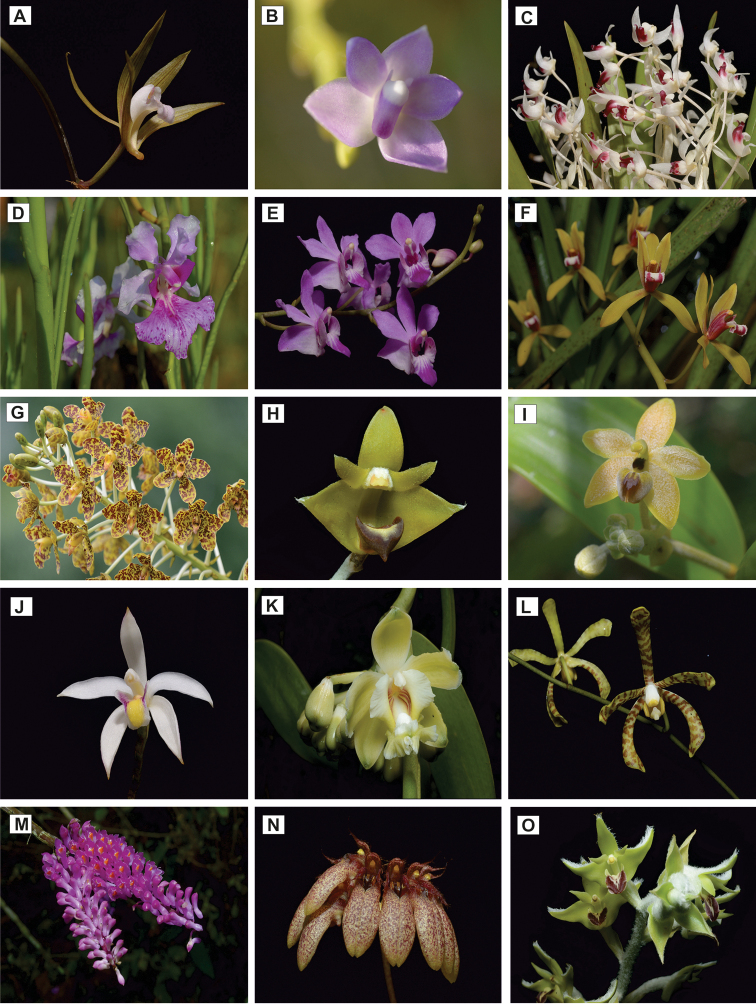
A selection of Orchidaceae species from Setiu Wetlands. **A***Ania penangiana***B***Thrixspermum amplexicaule***C***Pinalia atrovinosa***D***Papilionanthe hookeriana***E***Phalaenopsis pulcherrima***F***Cymbidium finlaysonianum***G***Grammatophyllum speciosum***H***Strongyleria pannea***I***Callostylis pulchella***J***Bromheadia finlaysoniana***K***Vanilla griffithii***L***Arachnis flos-aeris***M***Dendrobium secundum***N***Bulbophyllum trigonopus***O***Dendrolirium lasiopetalum*.

The Orchidaceae (Fig. 4) are well represented in SW, representing 23% of 245 orchid species recently reported in Terengganu (Besi et al. 2019). Thus, to date, there are 56 species of orchids found in SW from which 14 species were recorded by Siti Fatimah et al. (2015) and 42 represent new records in SW, mostly being recent collections by Dome Nikong. The highest number of orchid species in SW, as expected, are in the widespread genera *Bulbophyllum* and *Dendrobium*, similar to the results of Besi et al. (2019) in Tasik Kenyir logging sites. Both genera are found to be most abundant epiphytic orchids growing in disturbed and logged forests in which the weather and microclimate are favourable for growth and reproductive processes. However, orchid density is due in part to the severity of the disturbance in which highly disturbed logging sites harbour lower density than somewhat disturbed sites (Besi et al. 2019). Among the species recorded in SW, there are some that are exceptional. The orchid diversity in SW is enriched with the sighting of the uncommon *Papilionanthe hookeriana* that is confined to the freshwater swamp area of Tasik Berombak in SW. It usually coexists with shrubs and tall grasses for support (Pridgeon et al. 2014). On the other hand, the discovery of *Vanilla griffithii* in its uncharacteristic habitat of the BRIS forest signified its capability to thrive in xeric environment and supported its local genus distribution pattern suggested by Mohd Raffi et al. (2014) which was best described as constantly sparse, widespread and in many habitats.

As for the mangroves, there are about 33 exclusive mangrove species including three hybrids i.e. Sonneratia × hainanensis, Bruguiera × rhynchopetala, Rhizophora × annamalayana and four individuals of *Bruguiera hainesii* located at Pulau Layat (Razali et al. 2017). However, the mangroves in SW and on the east coast of Peninsular Malaysia, in general, are not so diverse and widely distributed as compared to the west coast because the former are exposed to the lagoons and rivers (Latiff and Faridah-Hanum 2014), and are also threatened by strong waves during monsoon months as well as anthropogenic activities e.g., many mangroves in SW had been uprooted to make way for aquaculture, shrimp ponds and constructions of infrastructures.

**Figure 5.**
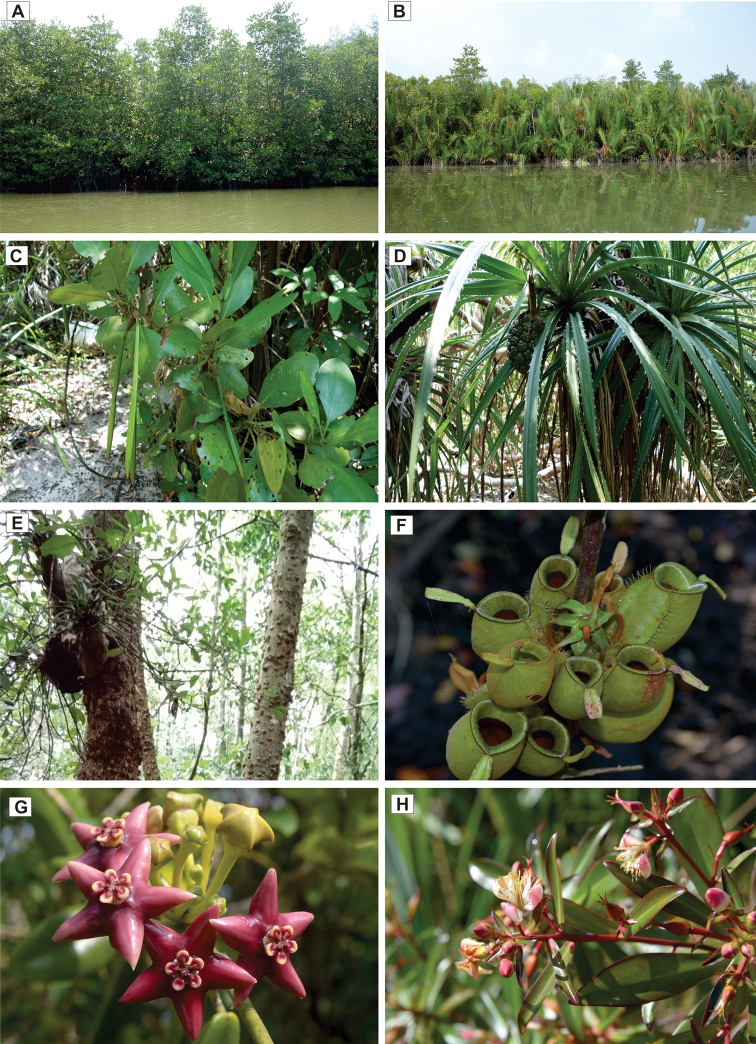
Different plant communities in Setiu Wetlands. **A** Mangrove plants **B** Nipa palm (*Nypa fruticans*) population **C***Ceriops zippeliana***D***Pandanus tectorius***E** Ant plant, *Hydnophytum formicarum* attached to *Bruguiera hainesii***F***Nepenthes ampullaria***G***Hoya coronaria***H***Ploiarium alternifolia*.

The relatively species rich profile of SW reflects on the interconnected forest types in SW which consists of different plant communities (Fig. 5) including beach, mangroves, peat swamp and freshwater swamp plants. Beach vegetation includes Casuarinaceae and Convolvulaceae and mixed mangroves plants such as the families Avicenniaceae, Lythraceae and Rhizophoraceae. Peat swamp plants can be found behind the mangrove belt and further inland, *Melaleuca* swamp forest dominates the waterlogged area associated with BRIS soil (Jamilah et al. 2015). On the other hand, the heath-like dune landscape established on the ridge areas of Setiu coast is characterised by stunted and low stature vegetation growing in a clumping pattern (Jamilah et al. 2014).The vegetation on the sandy and dry ridge is dominated by Myrtaceae family (*Melaleuca cajuputi*, *Baeckea frutescens*, *Rhodomyrtus tomentosa* and *Syzygium* spp.). Woody epiphytic shrubs (e.g., *Ficus deltoidea*) and herbaceous species such as orchids are adapted to grow underneath the clump on BRIS soil dune landscape (Jamilah et al. 2014). However, the natural ecosystem on BRIS soil ridge and swamps is becoming scarce and smaller in coverage due to various threats faced by the coastal ecosystem of SW. It has become more scattered and fragmented, resulting in difficulty in finding an area that could be a good representative of BRIS soil flora. Fragmentation and degradation also expose this natural ecosystem to the invasion of exotic invasive alien species, such as *Acacia mangium*, *A. auriculiformis* and their hybrids (Jamilah et al. 2014). It is predicted that without legal protection and authority commitment to conserve BRIS soil natural vegetation, it will soon be replaced by these alien species, particularly *Acacia* spp. Although the gazettement of BRIS soil habitat is still underway, land conversion in BRIS is rampant and to prevent further land uses, ecotourism activity is recommended. Therefore, the hope is that in the near future, BRIS soil habitat would be included in the next phases of State Park gazettement which will likely have a significant effect in ensuring the conservation of this unique habitat.

As for the wetland or swamp, a rainfed swamp with a water table is highest during the monsoon months. The swamp is dominated by *Melaleuca cajuputi* trees with larger diameter of *M. cajuputi* trees relative to trees growing on the drier ridge of BRIS soil, as it grows better in waterlogged conditions as compared to dry sites (Suzuki 1999). This *Melaleuca* swamp harbours carnivorous species of pitcher plants (*Nepenthes* spp.), sundews (*Drosera burmannii* in particular) and *Utricularia bifida* which are adapted to freshwater swamp. The hydrological contribution of patches of *Melaleuca* swamp as a seasonal wetland is worth exploring and the wetlands may provide a critical ecosystem service of mitigating floods, particularly in monsoon months in Terengganu (Jamilah et al. 2015). In addition, SW also harbours a large freshwater lake, locally known as Tasik Berombak. The water is contributed by rain and a few small river tributaries (Sathiamurthy 2015) and comprises BRIS soil with heath-like vegetation on its ridge, but is less rich than natural BRIS ecosystem. The lake is invaded by thick bush of *Hanguana malayana* and other aquatic and semi aquatic non vascular plants.

The high diversity of wild orchids and other potentially useful plant species on coastal habitat of SW is indeed a natural capital for SW State Park and furthermore, the habitat supports an option value, which could be tapped in the future as outlined in Total Economic Value (TEV) (Costanza et al. 1997). The biodiversity resources in SW can be managed sustainably to support the local community green economy as an alternative to unsustainable economic activities such as sand mining. The SW State Park will also be crucial to protect the critically endangered Painted Terrapin (*Batagur borneoensis* (Schlegel & Muller, 1844)) and to serve as a refuge for some 29 mammals, 161 birds and 36 reptiles and amphibians (WWF-Malaysia). Furthermore, it is also classified as an Important Bird Area (IBA) by Birdlife International.

**Figure 6.**
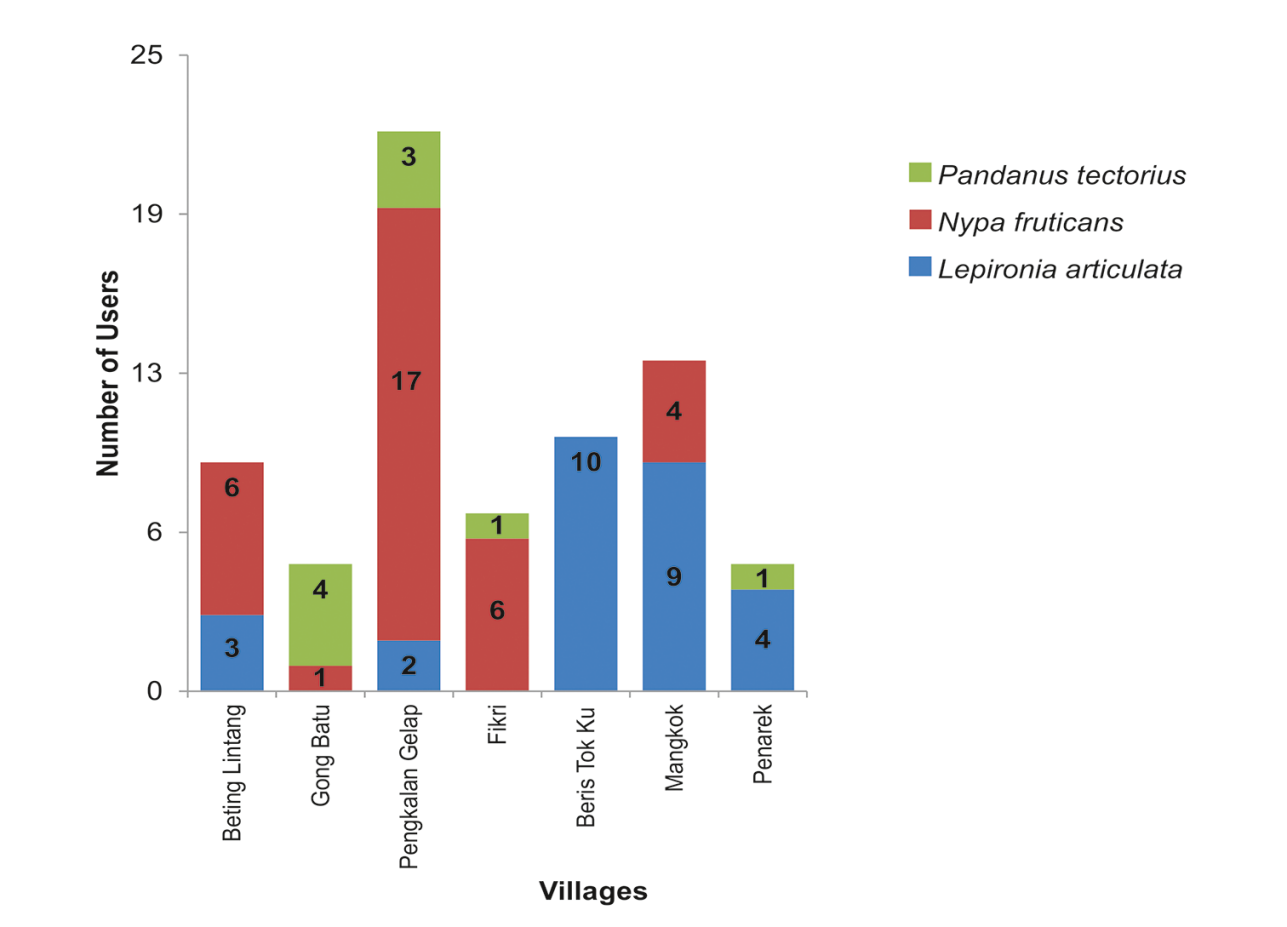
Number of various wild flora species users according to villages.

### Conservation status

Eight species have been classified as threatened species including one Critically Endangered (CR), *Bruguiera hainesii*, two Endangered (EN), *Anisoptera marginata* and *Pterocarpus indicus*, and five Vulnerable (VU) (*Avicennia rumphiana*, *Halophila beccarii*, *Intsia bijuga*, *Ternstroemia wallichiana* and *Vatica pauciflora*). The Critically Endangered, *B. hainesii* is only found in several localities in Southeast Asia including SW, and the lower estimates of mature individuals probably due to the low rates of propagation and germination (Polidoro et al. 2010). However, recent molecular analyses revealed that *B. hainesii* did not merit recognition of species as it has no unique haplotype/allele of its own but instead shared nuclear allele with *B. cylindrica* and *B. gymnorhiza*, and thus indicating the hybrid origin of *B. hainesii* (Ono et al. 2016). Five species partially met the classification thresholds under the threatened species category and therefore were listed as Near Threatened i.e. *Cycas edentata*, *Myristica lowiana*, *Olax scandens*, *Phoenix paludosa*, *Sonneratia ovata*, while 155 species are regarded either as Least Concern (LC) or Data Deficient (DD). However, about half of the vascular plants (59%) occurring in SW have not been assessed and categorised under the IUCN Red List of Threatened Species.

For the Malaysia Plant Red List, two species have been classified as threatened species, namely *Anisoptera marginata* (EN) and *Anodendron candolleanum* (VU). There were nine species listed as Near Threatened i.e. *Cycas edentata*, *Cerbera odollam*, *Cerbera manghas*, *Vatica pauciflora*, *Drosera burmannii*, *Xylocarpus moluccensis*, *Horsfieldia irya*, *Myristica lowiana*, *Olax scandens* while 32 species were listed as Least Concern (LC). The other 369 species have not been assessed and categorised under the Malaysia Plant Red List but are available in MyBIS. On the other hand, there were 55 species (13%) listed under CITES of which 49 species were from Orchidaceae, three from Nepenthaceae, two from Ebenaceae and one from Cycadaceae. Almost all the orchids recorded (89%) in SW are listed in CITES. About 30,000 plant species have been listed and protected by CITES against over-exploitation through international trade of which more than half of the species assessed are orchids and cacti.

### Wild flora based livelihoods in SW

**Figure 7.**
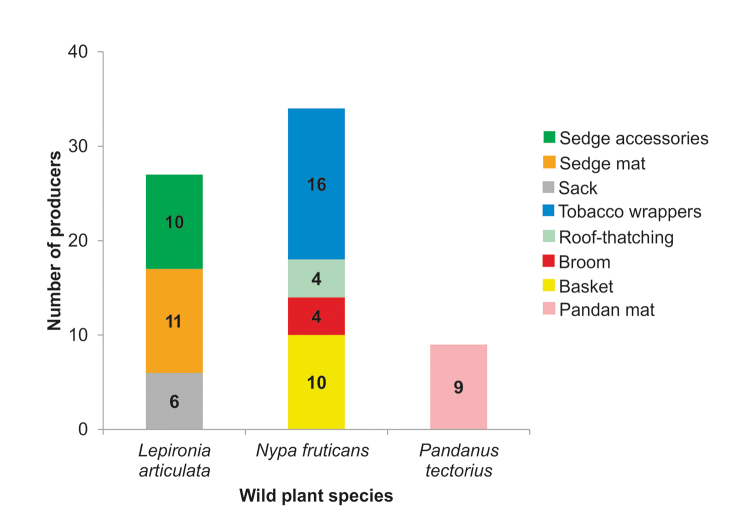
Number of producers based on type of products made from wild flora.

We found that in the SW, the local households’ utilisation mainly focused on three species, namely *Nypa fruticans* (nypa), *Lepironia articulata* (Blue-grey sedge) and *Pandanus tectorius* (Sea Pandan, Sea screwpine). Figure 6 shows the number of flora user households based on the species utilised in each village. *Nypa fruticans* records the highest number of users with 34 households from five out of seven villages. *Lepironia articulata* is a close second, with recorded utilisation in 28 households in five villages as well. Meanwhile *P. tectorius* is the least utilised of the three species with only nine user households in total from four villages. The wide use of *N. fruticans* coincides with the highest variety of products that can be made using its various plant parts (see Fig. 7). The nypa palm is the most versatile wild plant among the three as different parts of the plant are used to make different kinds of products. For example, the young leaves are used to make tobacco wrappers, its dried midrib is weaved into baskets, while mature fronds with leaves are used to make roof-thatch. The midribs of the nypa, which are unsuitable for weaving lekar baskets, are used to make brooms. Due to this, nypa is the most preferred plant species used in SW. Its utilisation is well documented in Malaysia (see Latiff 2009; Tsuji et al. 2011). It is not only an important wild resource for the Malays but also for indigenous tribes such as the Mah Meri who use nypa leaves to produce decorative items for spirit huts, altars, homes and dancers (Baba et al. 2013).

As for *Lepironia articulata*, although it can be used to make similar types of products as those made using *Pandanus tectorius*, i.e. mats and bags, its livelihood-based utilisation in Malaysia appears less recorded compared to the latter. Instead, there appears to be more documentation on its utilisation in grey water treatment (see Sim et al. 2008; Wurochekke et al. 2014). However, its utilisation is significant for the users in SW, as it supplemented up to 45% of their monthly household income and represents a strong cultural link to the local tradition for the users who are mostly exclusive (not using other flora resource) users of this resource. The 11 users from SW produced mats, six users made sacks, while 10 users made accessories’ items such as hats and bags. Our findings suggest that current utilisation is at a sustainable level thanks to the user’s knowledge about the ecology of these plants. Its use therefore poses no threat to the integrity of the state park. According to MacDonald (2009), *L. articulata* is listed among eight major species that are commonly used for weaving activities by the Plant Resources of South-East Asia (PROSEA) (Brink and Escobin 2003) due to their high suitability as a raw material for weaving activities, in particular their toughness, plasticity, sustainable strength and impermeability after being dried (Truyen et al. 2014).

**Figure 8.**
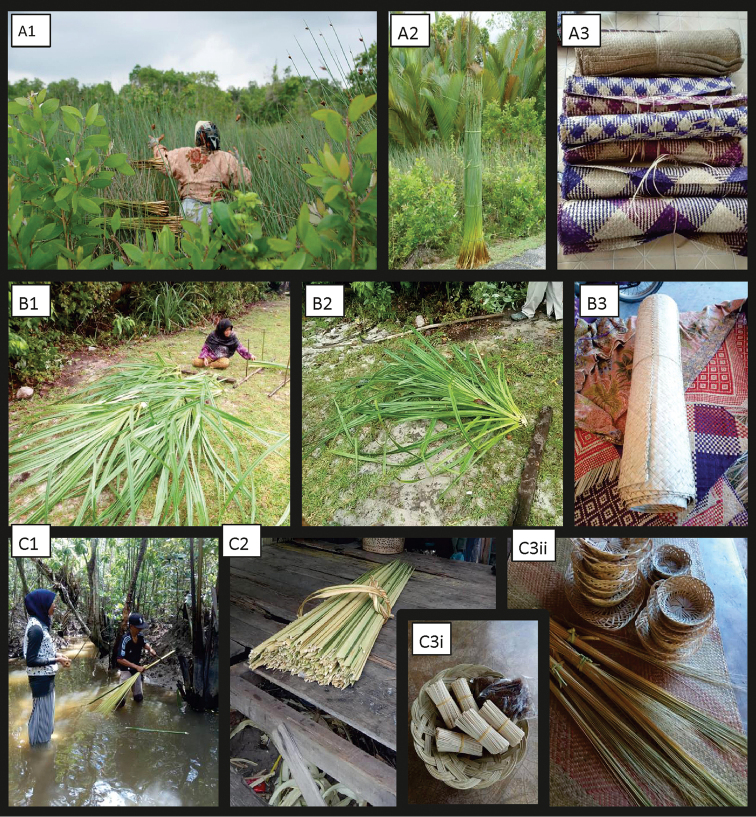
Utilisation of *Lepironia articulata* (**A1–3**), *Pandanus tectorius* (**B1–3**), and *Nypa fruticans* (**C1–3ii**) in Setiu Wetlands. From left to right **1** Harvesting **2** Part used **3** Finished products.

The utilisation of *L. articulata* has been documented in other countries like Indonesia, Vietnam, Thailand and China where this plant is used to make handicraft or household materials such as bags, mats, baskets, and hats (Domyos and Te-Chato 2013; Truyen et al. 2014). Whereas *P. tectorius* is only used to make one type of product, i.e. mats by nine users in SW, although other types of handicrafts used to be made in the past. Indeed, while pandan mats are produced in various parts of Malaysia (Ismail and Nawawi 2011; Baba et al. 2013) the quality of pandan mats produced by Terengganu weavers is of excellent quality (Ismail and Nawawi 2013). Therefore, it is highly probable that the weavers in SW could also produce a variety of products (Fig. 8), just as the weavers of Mah Meri tribe who are well known for producing varied, exquisite handicrafts such as purses, pouches, mats and baskets in Pulau Carey, Selangor (Baba et al. 2013). However, there needs to be a steady market demand that guarantees a good income stream, which is provided to the Mah Meri weavers by the Gerai OA, an NGO that helps market their products through fairs and online marketing.

## Conclusions

Our survey indicates that the nine connected habitats in SW are relatively rich in vascular plants, harbouring nearly 20% of Peninsular Malaysia wetland flora. The current checklist is far from complete as additional species will likely be found with wider sampling coverage and additional systematic inventories. The utilisation of plant resources for the livelihood of coastal communities in SW is still significant for the three main species used in the area (*Nypa fruticans*, *Lepironia articulata* and *Pandanus tectorius*). Local communities play an important role in the sustainability of SW, so it is essential to understand their dependence on the intricate network of wetland ecosystems and their plant species to ensure that they are not overlooked in the management plans of the Setiu Wetlands State Park.

## Appendix 1

**Table:** Checklist of vascular plants from Setiu Wetlands, Terengganu, Malaysia. The habitat for all species are abbreviated as MSF = Mangrove Swamp Forest; PSF = Peat Swamp Forest; RV = Riparian Vegetation; LF = Lowland Forest; HV= Heath vegetation including CDF= Coastal Dunes Forest; BV = Beach Vegetation; DV = Disturbed Vegetation; FSF = Freshwater Swamp Forest; and BRIS including *Melaleuca* vegetation (MV=*Melaleuca* vegetation). Six categories in the conservation status, EN: Endangered, VU: Vulnerable, NT: Near Threatened, LC: Least Concern, DD: Data Deficient, NE: Never Evaluated.

Family	Species	Local Name	Life Form	Habitat	IUCN Status	Malaysia Red List/MyBIS
**LYCOPHYTES**						
Lycopodiaceae	*Lycopodiella cernua* (L.) Pic.Serm.	Sorok-sorok	Fern	RV, DV	LC	NE
**FERNS**						
Aspleniaceae	*Asplenium longissimum* Blume	-	Fern	LF	NE	NE
	*Asplenium nidus* L.	Paku Sarang Burung, Daun Semun, Paku Langsuir, Paku Langsuyar, Paku Pandan, Paku Sakat, Rumah Langsuyar	Fern	LF	NE	NE
Blechnaceae	*Blechnum indicum* Burm.f.	-	Fern	CDF	NE	NE
	*Stenochlaena palustris* (Burm.f.) Bedd.	Paku Miding	Fern	LF	NE	NE
Davalliaceae	*Davallia denticulata* (Burm.f.) Mett. ex Kuhn.	Paku Tertutup, Paku Terutup	Fern	FSF, RV, DV	NE	LC
	*Davallia solida* (Forst.) Sw.	-	Fern	BV	NE	NE
Dennstaedtiaceae	*Pteridium esculentum* (G.Forst.) Cockayne	-	Fern	LF	NE	NE
Lygodiaceae	*Lygodium flexuosum* (L.) Sw.	Paku Ribu-ribu, Akar Sidin, Darai Paya	Fern	LF	LC	LC
	*Lygodium microphyllum* (Cav.) R.Br.	Paku Ribu-ribu, Selada	Fern	DV	LC	LC
Nephrolepidaceae	*Nephrolepis auriculata* (L.) Trimen	Paku Hitam	Fern	DV	DD	NE
	*Nephrolepis biserrata* (Sw.) Schott	Paku Hitam, Paku Larat, Paku Uban	Fern	DV	LC	LC
Polypodiaceae	*Drynaria quercifolia* (L.) J.Sm.	Paku Sakat Tupai, Sakat Laipang, Daun Kelapa Tupai, Daun Kepala Tupai	Fern	LF	LC	LC
	*Goniophlebium percussum* (Cav.) Wagner & Grether	Paku Pakis	Fern	LF	LC	LC
	*Phymatosorus cuspidatus* (D.Don) Pic.Serm.	Paku Pakis	Fern	LF, DV	DD	NE
	*Phymatosorus scolopendria* (Burm.f.) Pic.Serm.	Paku Wangi, Sakat Hitam	Fern	LF, DV	DD	NE
	*Pyrrosia lanceolata* (L.) Farw.	Bulu Ayam, Sakat Batu, Tetumpang	Fern	BV,CDF	LC	LC
	*Pyrrosia piloselloides* (L.) M.G.Price	Duit-duit, Sakat Ribu-ribu, Sisik Naga	Fern	LF, MSF	LC	LC
	*Pyrrosia longifolia* (Burm.f.) C.V.Morton	Sakat, Suloi	Fern	LF, FSF, DV	LC	LC
Psilotaceae	*Psilotum nudum* (L.) P.Beauv.	-	Fern	CDF	NE	NE
Pteridaceae	*Acrostichum aureum* L.	Piai Raya, Paku Larat, Paku Laut	Fern	MSF	LC	LC
	*Acrostichum speciosum* Willd.	Piai Lasa	Fern	MSF	LC	LC
Salviniaceae	*Salvinia molesta* D.S.Mitch.	Kiambang	Fern	FSF	NE	NE
Schizaeaceae	*Schizaea dichotoma* (L.) Sm.	Janggut keli, Payung Ali, Misai Rimau, Paku Cakar Ayam	Fern	CDF	LC	NE
**GYMNOSPERMS**						
Cycadaceae	*Cycas edentata* De Laub.	Pokok Sakat	Tree	CDF	NT	NT
Gnetaceae	*Gnetum cuspidatum* Blume	Melinjau akar	Climbing Shrub	LRW	LC	NE
	*Gnetum gnemon* L.	Melinjau/Cokok	Tree	BV	LC	NE
**DICOTYLEDONS**						
Acanthaceae	*Acanthus ebracteatus* Vahl	Jeruju Putih	Shrub	MSF, RV, LF	LC	NE
	*Acanthus ilicifolius* L.	Jeruju Hitam	Shrub	MSF	LC	NE
	*Ruellia tuberosa* L.	-	Terrestrial herb	DV	NE	NE
	*Thunbergia fragrans* Roxb.	Akar Patuk Tuau, Akar Sebiak, Kacang Akar, Kelemai Merah, Sambung Nyawa, Tunbergia Putih	Terrestrial herb	DV	NE	NE
Anacardiaceae	*Anacardium occidentale* L.	Gajus, Jambu Golok, Jambu Monyet,	Tree	CDF	NE	NE
Anacardiaceae	*Buchanania arborescens* (Blume) Blume	Otak Udang Tumpul, Otak Udang, Katak Udang, Ketak Udang, Puah Pipit, Puan, Terentang Tikus	Tree	RV, CDF	NE	NE
	*Buchanania sessilifolia* Blume	Otak Udang Tumpul	Tree	LF	DD	NE
	*Campnosperma coriaceum* (Jack) Hallier f. ex Steenis	Terentang, Terentang Kelintang	Tree	SF, FSF	NE	NE
	*Campnosperma squamatum* Ridl.	Terentang, Terentang Daun Kecil,	Tree	SF	NE	NE
	*Gluta velutina* Blume	Rengas, Rengas air	Tree	SF, RV, CDF	NE	NE
	*Mangifera griffithii* Hook.f.	Asam Raba, Asam Rawa, Rawa	Tree	LF	NE	NE
	*Mangifera pentandra* Hook.f.	Pauh, Mangga, Mangga Air, Mangga Dodol, Mempelam Bemban	Tree	LF	DD	NE
	*Gluta wallichii* (Hook.f) Ding Hou	Rengas	Tree	PSF	DD	NE
Annonaceae	*Annona glabra* L.	Nona Licin	Tree	MSF	NE	NE
Ancistrocladaceae	*Ancistrocladus tectorius* (Lour.) Merr.	Akar Julong Hitam, Jejulong Akar	Shrub	LF	NE	LC
	*Alyxia reinwardtii* Blume	Palusari	Climbing shrub	LF, PSF	NE	LC
	*Anodendron candolleanum* Wight	Akar kikat, Akar nirwali	Climbing shrub	PSF, RV	NE	VU
	*Calotropis gigantea* (L.) W.T.Aiton	Remingu	Shrub	CDF, BV	NE	NE
	*Cerbera odollam* Gaertn.	Pong-pong, Buta-buta	Tree	SF	NE	NE
	*Cerbera manghas* L.	Pong-pong, Pong Pong Pong, Buta-buta, Nyan	Tree	MSF, BV	NE	NT
	*Dischidia major* (Vahl) Merr.	Akar Bano, Akar Kul	Epiphytic herb	BV	NE	NE
	*Dischidia nummularia* R.Br.	Daun Pitis Kecil	Epiphytic herb	FSF	NE	NE
	*Finlaysonia obovata* Wall.	Kalak kambing, Pelir Kambing	Climbing shrub	MSF	NE	NE
	*Hoya carnosa* (L.f.) R.Br.	Akar Banok Jantan	Climbing shrub	BV	NE	NT
	*Hoya coronaria* Blume	Akar Setebal	Epiphytic shrub	MSF	NE	NE
	*Hoya coriacea* Blume	Akar Setebal	Epiphytic shrub	MSF	NE	NE
Ancistrocladaceae	*Hoya diversifolia* Blume	Akar Setebal	Epiphytic shrub	MSF	NE	NE
	*Hoya verticillata* (Vahl) G.Don	Akar Setebal	Epiphytic shrub	MSF	NE	NE
	*Parsonsia alboflavescens* (Dennst.) Mabb.	-	Climbing shrub	MSF, PSF, BV	NE	NE
	*Tylophora flexuosa* R.Br.	Akar Banok Jantan	Climbing shrub	MSF	NE	NE
Aquifoliaceae	*Ilex cymosa* Blume	Mensirah, Mensirah Puteh	Tree	MSF	NE	NE
Araliaceae	*Arthrophyllum diversifolium* Blume	Tumbuh Kelapa	Tree	LF	NE	NE
	*Schefflera elliptica* (Blume) Harms.	Cenama Gajah	Climbing shrub	MSF	NE	NE
Apocynaceae	*Alstonia pneumatophora* Baker ex Den Berger	Pulai Paya	Tree	FSF	LC	NE
	*Catharanthus roseus* (L.) G.Don	Kemunting Cina	Shrub	CDF, MSF	NE	NE
	*Sarcolobus globosus* Wall.	Buah Pitis	Climbing shrub	MSF	NE	NE
	*Secamone elliptica* R.Br.	-	Shrub	DV	NE	NE
Asteraceae	*Melanthera biflora* (L.) Wild	Serenai Laut, Seremai, Serenah, Sunai Laut	Climbing herb	MSF, BV	NE	NE
	*Mikania micrantha* Kunth	Selaput Tunggul	Climbing herb	DV	NE	NE
	*Pluchea indica* (L.) Less	Beluntas	Shrub	MSF	NE	NE
	*Sphagneticola trilobata* (L.) Pruski	-	Terrestrial herb	DV	NE	NE
	*Synedrella nodiflora* (L.) Gaertn.	-	Terrestrial herb	DV	NE	NE
	*Tridax procumbens* L.	Butang Baju	Terrestrial herb	DV	NE	NE
Avicenniaceae	*Avicennia alba* Blume	Api-api Putih, Api-api Hitam	Tree	MSF, RV	LC	NE
	*Avicennia officinalis* L.	Api-api Ludat, Api-api, Api-api Sudu	Tree	MSF	LC	NE
	*Avicennia rumphiana* Hallier f.	Api-api Bulu	Tree	MSF	VU	NE
Bignoniaceae	*Dolichandrone spathace*a (L.f.) K.Schum.	Tui, Poko Kulo, Tuj, Kulok	Tree	MSF	LC	NE
Bonnetiaceae	*Ploiarium alternifolium* (Vahl) Melchior	Riang-riang	Tree	FSF, DV	NE	LC
Calophyllaceae	*Calophyllum inophyllum* L.	Bintangor laut	Tree	CDF	LC	NE
	*Calophyllum rupicola* Ridl.	Bintangor	Tree	MSF, CDF	NE	NE
	*Calophyllum sclerophyllum* Vesque	Bitangor Jangkang	Tree	PSF	NE	NE
	*Mesua ferruginea* (Pierre) Kosterm.	Sembawang	Tree	PSF	NE	NE
Casuarinaceae	*Casuarina equisetifolia* L.	Rhu, Ru	Tree	CDF, BV	NE	NE
Celastraceae	*Gymnosporia littoralis* (Backer) Jordaan	-	Shrub	CDF	NE	NE
	*Salacia chinensis* L.	Akar pelanduk	Climbing shrub	MSF	NE	NE
Chrysobalanaceae	*Licania splendens* (Korth.) Prance	Nyalas	Tree	PSF, LF	LC	LC
	*Parastemon urophyllus* (Wall.ex A.DC.) A.DC.	Malas Siangus	Tree	CDF, BV	NE	NE
Clusiaceae	*Garcinia hombroniana* Pierre	Beruas	Tree	CDF, BV	NE	NE
	*Garcinia nigrolineata* Planch.ex T.Anderson	Beruas	Tree	CDF	NE	NE
	*Garcinia brevirostris* Scheff.	Lulai, Kandis	Tree	CDF	NE	NE
	*Garcinia parvifolia* (Miq.) Miq.	Beruas	Tree	FSF	NE	NE
Combretaceae	*Lumnitzera littorea* (Jack) Voigt	Teruntum Merah	Tree	MSF	LC	NE
	*Lumnitzera racemosa* Willd.	Teruntum Putih, Teruntum Bunga Putih	Tree	MSF	LC	NE
	*Combretum tetralophum* C.B.Clarke	-	Climbing shrub	PSF	NE	NE
	*Terminalia catappa* L.	Ketapang	Tree	MSF, CDF, BV	NE	NE
Convolvulaceae	*Ipomoea cairica* (L.) Sweet	Seri pagi jalar	Terrestrial herb	CDF,BV	LC	NE
	*Ipomoea pes-caprae* (L.) R.Br	Tapak Kuda, Seri pagi	Terrestrial herb	CDF, DV, BV	NE	NE
Dilleniaceae	*Dillenia suffruticosa* (Griff.) Martelli.	Simpoh Air	Tree	LF,DV	NE	NE
	*Tetracera indica* (Christm. & Panz.) Merr.	Akar Mempelas Licin, Akar Mempelas, Mempelas, Mempelas Minyak, Mempelas Paya	Climbing shrub	LF,DV	NE	NE
	*Tetracera scandens* (L.) Merr.	Akar Mempelas	Climbing shrub	LF,DV,BV	NE	NE
Dioscoreaceae	*Tacca leontopetaloides* (L.) Kuntze	Lekir Pasir	Terrestrial herb	CDF, BV	LC	NE
Dipterocarpaceae	*Anisoptera marginata* Korth.	Mersawa Paya	Tree	PSF	EN	EN
	*Vatica pauciflora* Blume	Resak raya	Tree	MSF	VU	NT
Droseraceae	*Drosera burmannii* Vahl	-	Terrestrial herb	LF	LC	NT
Ebenaceae	*Diospyros ferrea* (Willd.) Bakh.	Buey, Kayu Arang, Kayu Arang	Tree	MSF	NE	NE
	*Diospyros lanceifolia* Roxb.	Arang	Tree	LF	NE	NE
	*Diospyros maingayi* (Hiern) Bakh.	Kayu Arang, Siangus, Merpinang Daun Besar	Tree	PSF, LF	NE	NE
Elaeocarpaceae	*Elaeocarpus macrocerus* (Turcz.) Merr.	Mendong	Tree	FSF	NE	NE
	*Elaeocarpus mastersii* King	Mendong	Tree	LF, FSF, PSF	NE	LC
	*Elaeocarpus petiolatus* (Jack) Wall	Mendong	Tree	LF	NE	LC
Ericaceae	*Styphelia malayana* (Jack) Spreng.	Choreng atap, Chuchur Atap, Maki China, Tasek Timbul	Tree	CDF,BV	NE	NE
	*Vaccinium littoreum* Miq.	Inai batu	Tree	BV	NE	NE
Erythroxylaceae	*Erythroxylum cuneatum* (Miq.) Kurz.	Cinta Mula	Tree	CDF, BV	NE	NE
Euphorbiaceae	*Excoecaria agallocha* L.	Buta-buta, Buta-buta, Bebuta, Betak-betak	Tree	MSF	LC	NE
	*Macaranga hypoleuca* (Rchb.f. & Zoll.) Müll.Arg.	Mahang Putih	Tree	LF,DV	NE	NE
	*Macaranga laciniata* Whitmore & Airy Shaw.	Mahang	Tree	LF,DV	NE	NE
	*Shirakiopsis indica* (Willd.) Esser	Gurah	Tree	MSF	LC	NE
	*Suregada multiflora* (A.Juss.) Baill.	Merlimau, Limau Hantu	Tree	LF	NE	NE
Fabaceae	*Aganope heptaphylla* (L.) Polhill	Ketui Besar, Omis omis	Climbing shrub	MSF, BV	NE	NE
	*Archidendron clypearia* (Jack) I.C.Nielsen	Petai Kera	Tree	PSF, DV	NE	NE
	*Caesalpinia bonduc* (L.) Roxb.	Gorek	Climbing shrub	CDF, BV	NE	NE
	*Caesalpinia crista* L.	Akar Kuku Tupai	Climbing shrub	MSF	NE	NE
	*Canavalia rosea* (Sw.) DC.	Kekacang Laut	Terrestrial herb	BV	NE	NE
	*Cynometra ramiflora* L.	Katak Puru	Tree	MSF	NE	NE
	*Dalbergia candenatensis* (Dennst.) Prain	Akar Kait, Api-api Jambu,	Climbing shrub	MSF	NE	NE
	*Dendrolobium umbellatum* (L.) Benth.	Petai laut, Dedulang, Petai belalang, Petai laut	Shrub	MSF	NE	NE
	*Derris trifoliata* Lour.	Ketui, Tuba laut, Ketui, Salang, Selang, Setui	Climbing shrub	MSF	NE	NE
	*Desmodium adscendens* (Sw.) DC.	Sisik Naga	Terrestrial herb	PSF, DV	LC	NE
	*Desmodium heterophyllum* (Willd.) DC.	Sisik Naga	Terrestrial herb	LF, DV	NE	NE
	*Desmodium triflorum* (L.) DC.	Sisik Naga	Terrestrial herb	DV	LC	NE
Fabaceae	*Intsia bijuga* (Colebr.) Kuntze	Ipil, Merbau Ipil, Merbau Changkat, Merbau laut	Tree	MSF	VU	NE
	*Mimosa pudica* L.	Semalu	Terrestrial herb	DV	LC	NE
	*Ormosia sumatrana* (Miq.) Prain	Sepit-sepit	Tree	LF	NE	NE
	*Peltophorum pterocarpum* (DC.) K.Heyne	Jemerlang	Tree	MSF, BV	NE	NE
	*Pongamia pinnata* (L.) Pierre	Mempari	Tree	MSF, BV	LC	NE
	Pongamia pinnata (L.) Pierre var. xerocarpa (Hassk.) Alston	Malapari	Tree	RV,LF		
	*Pterocarpus indicus* Willd.	Angsana	Tree	CDF	VU	NE
	*Senna alata* (L.) Roxb.	Gelenggang	Shrub	LSF,DV	NE	NE
	*Senna occidentalis* (L.) Link	Gelenggang Pasir	Shrub	DV	NE	NE
	*Tamarindus indica* L.	Asam Jawa	Tree	LF	LC	NE
Gentianaceae	*Cyrtophyllum fragrans* (Roxb.) DC.	Tembusu	Tree	FSF, DV	NE	NE
	*Fagraea auriculata* Jack	Pelir Musang	Tree	CDF	NE	NE
	*Fagraea racemosa* Jack	Kahwa Hutan	Tree	LSF,DV	NE	NE
	*Fagraea fragrans* Roxb.	Tembusu	Tree	FSF, DV	LC	NE
Goodeniaceae	*Scaevola taccada* (Gaertn.) Roxb.	Ambong-ambong	Shrub	BV	NE	LC
Hemerocallidaceae	*Dianella ensifolia* (L.) DC.	Siak-siak, Akar Siak, Benjuan, Jamaka, Lenjuang, Meroyan Bangkai, Setagit, Senjuang	Terrestrial herb	LF	NE	NE
Hypericaceae	*Cratoxylum arborescens* (Vahl) Blume	Geronggang	Tree	PSF, DV	LC	LC
Lamiaceae	*Volkameria inermis* L.	Lampin Budak, Gambir Laut, Pawan, Tulang-tulang	Shrub	MSF	NE	NE
	*Gmelina elliptica* Sm.	Bulangan	Shrub	DV	NE	NE
	*Premna serratifolia* L.	Buas-buas, Bangkung Kayu, Sarunai,	Shrub	MSF, BV	NE	NE
	*Vitex pinnata* L.	Leban	Tree	DV	NE	NE
	*Vitex rotundifolia* L.f.	Langundi	Shrub	BV	NE	NE
	*Vitex trifolia* L.	Halban, Lagundi	Tree	MSF	NE	NE
Lauraceae	*Cassytha filiformis* L.	Cemar batu	Parasitic herb	CDF, BV	NE	NE
	*Neolitsea zeylanica* (Nees) Merr.	Teja pasir	Tree	CDF	NE	NE
	*Phoebe grandis* (Nees) Merr.	Medang	Tree	LF	NE	NE
Lentibulariaceae	*Utricularia bifida* L.	-	Herbaceous	PSF	LC	NE
Loranthanceae	*Dendrophthoe pentandra* (L.) Miq.	Dedalu	Parasitic shrub	LF	NE	NE
Lecythidaceae	*Barringtonia asiatica* (L.) Kurz.	Putat Laut, Butong, Butun, Butung	Tree	BV	LC	LC
	*Barringtonia racemosa* (L.) Spreng.	Putat Sungai, Putal Kedul, Putat Air, Putat Ayam, Putat Darat, Putat Kampung, Putat Padi, Putat Rambai, Putat Sawah	Tree	MSF	NE	NE
Linaceae	*Indorouchera griffithiana* (Planch.) Hallier f.	Akar Ipoh	Climbing shrub	LF, PSF	NE	NE
Lythraceae	*Lagerstroemia speciosa* (L.) Pers.	Bungor, Bungor Biru, Bungor Rya, Tibabah	Tree	DV	NE	NE
	*Sonneratia alba* Sm.	Perepat, Pauh Kijang	Tree	MSF	LC	NE
	*Sonneratia caseolaris* (L.) Engl.	Berembang, Perapat, Perapat Laut, Perepat	Tree	MSF	LC	NE
	*Sonneratia* x *hainanensis* W.C.Ko	Gedabu Hibrid	Tree	MSF	DD	NE
	*Sonneratia lanceolata* Blume	Berembang Putih,	Tree	MSF	LC	NE
	*Sonneratia ovata* Backer	Gedabu, Kedabu, Rogam	Tree	MSF	NT	NE
Malpighiaceae	*Tristellateia australasiae* A.Rich.	-	Climbing shrub	MSF	NE	NE
Malvaceae	*Brownlowia argentata* Kurz.	Durian Laut	Tree	MSF,BV	DD	NE
	*Commersonia bartramia* (L.) Merr.	Angkut Besi	Tree	LF,DV	LC	NE
	*Heritiera littoralis* Aiton	Dungun, Bayur Laut, Buah Pelir Kambing, Atun Laut	Tree	MSF	LC	NE
	*Talipariti tiliaceum* (L.) Fryxell	Baru-baru Laut, Bebaru, Bebaru Laut	Tree	MSF	LC	NE
	*Thespesia populnea* (L.) Sol. ex Correa	Bebaru, Baru Laut, Buah Keras Laut	Tree	MSF	LC	NE
	*Sida acuta* Burm.f.	Kelulut Putih	Terrestrial herb	DV	NE	NE
	*Urena lobata* L.	Pulut-pulut, Pepulut, Pulut Lembu	Terrestrial herb	DV	DD	NE
Melastomataceae	*Melastoma malabathricum* L.	Senduduk, Kenduduk	Shrub	LF	DD	NE
	*Memecylon caeruleum* Jack	Dali Dedali, Delek Jambu	Tree	LF	LC	LC
	*Memecylon edule* Roxb.	Delek Air, Nipis Kulit, Delek Ayer	Tree	LF	LC	LC
Meliaceae	*Xylocarpus granatum* J.Koenig	Nyireh Bunga, Nyireh	Tree	MSF	LC	LC
	*Xylocarpus moluccensis* (Lam.) M.Roem.	Nyireh Batu	Tree	MSF	LC/NT	LC
Menyanthaceae	*Nymphoides indica* (L.) Kuntze	Telipot	Aquatic herb	DV	LC	NE
Moraceae	*Ficus deltoidea* Jack	Mas Cotek, Ara, Serapat Angin, Telinga Beruk	Shrub	LF	DD	NE
	*Ficus microcarpa* L.F.	Beringin, Ara Jejawi, Jawi Jawi	Tree	MSF	LC	NE
	*Ficus sundaica* Blume	Ara Bertih, Ara Punai	Tree	LF	DD	NE
Myrtaceae	*Baeckea frutescens* L.	Cucur Atap, Tuturun Atap, Rempah Rempah	Tree	PSF	LC	NE
	*Melaleuca cajuputi* Powell	Gelam Putih, Kayu Putih	Tree	PSF, MSF	LC	NE
	*Rhodamnia cinerea* Jack	Mempoyan, Mempoyan Bukit, Mengkoyan Pinang	Tree	LF	LC	NE
	*Rhodomyrtus tomentosa* (Aiton) Hassk.	Kemunting, Lidah Katak Laut	Shrub	BV	LC	NE
	*Syzygium antisepticum* (Blume) Merr. & L.M.Perry	Kelat Tikus, Gelam Tikus, Kelat Gelam	Tree	LF	NE	NE
	Syzygium densiflora var. angustifolia Ridl.	-	Tree	LF	DD	NE
	*Syzygium grande* (Wight) Walp. & Wight	Jambu laut, Jambu Air Laut, Kelat Jambu Laut	Tree	CDF	NE	NE
	*Syzygium oblatum* (Roxb.) Wall. ex A.M. Cowan & Cowan	Kelat Kecham	Tree	LF, PSF	DD	NE
	*Syzygium incarnatum* (Elmer) Merr. & L.M.Perry	Kelat Kertas, Kulat Gelam	Tree	LF, PSF	DD	NE
	*Syzygium leucoxylon* Korth.	Kelat Putih	Tree	PSF	DD	NE
	*Syzygium pyrifolium* (Blume) DC.	Kelat Putih, Kelat Lapis	Tree	LF, PSF	NE	NE
	*Syzygium palembanicum* Miq.	Jambu, Kelat	Tree	LF	NE	NE
Myrtaceae	*Syzygium syzygioides* (Miq.) Merr. & L.M.Perry	Kelat Hitam	Tree	LF	NE	NE
	*Syzygium zeylanicum* (L.) DC.	Kelat Gelam, Jambu, Gelam Tikus, Kelat Nenasi, Ubah Gelam	Tree	LF, FSF	NE	NE
Myricaceae	*Morella esculenta* (Buch.-Ham. ex D.Don) I.M.Turner	Telur Cicak, Kesami, Keteng, Lenketing	Tree	FSF	LC	LC
Myrisinaceaae	*Aegiceras corniculatum* (L.) Blanco	Teruntun, Kacang Kacang, Kuku Helang	Tree	MSF	LC	NE
	*Rapanea porteriana* (Wall. ex A.DC.) Mez	Dedahruang	Tree	MSF	NE	NE
Myristicaceae	*Horsfieldia irya* (Gaertn.) Warb.	Pianggu, Penarahan	Tree	MSF, FSF	LC	NT
	*Knema conferta* (King) Warb.	Penarahan Hitam	Tree	LF	LC	NE
	*Knema globularia* (Lamk.) Warb.	Penarahan Padi, Chendarah Padi	Tree	LF	NT	NE
	*Myristica lowiana* King	Penarahan arang, Penarah Arang Gambut	Tree	PSF	NT	NT
Nepenthaceae	*Nepenthes ampullaria* Jack	Periok Kera	Climbing shrub	LF, FSF	LC	NE
	*Nepenthes gracilis* Korth.	Periok Kera	Climbing shrub	PSF	LC	NE
	*Nepenthes mirabilis* (Lour.) Druce	Periok Kera	Climbing shrub	PSF,LF	LC	NE
Ochnaceae	*Brackenridgea hookeri* (Planch.) A.Gray	Bunga Kelat Merah, Mata Ketam, Kayu Luru	Tree	LF	LC	NT
Olacaceae	*Olax scandens* Roxb.	Kodak Aching, Meribut	Shrub	DV	NT	NE
Oleaceae	*Olea brachiata* (Lour.) Merr.	Menserah	Tree	BV	NE	NE
Opiliaceae	*Champereia manillana* (Blume) Merr.	Chemperai	Tree	FSF	LC	NE
	*Cansjera rheedei* J.F.Gmel.	Chemperai Akar	Shrub	BV	NT	NE
Passifloraceae	*Passiflora foetida* L.	Buah Letup, Buah Tikus, Pokok Lang Bulu, Timun Denfdang, Timun Hutan	Climbing herb	RV, DV	NE	NE
Peraceae	*Chaetocarpus castanocarpus* (Roxb.) Thwaites	Membatu, Bebatu, Bedik	Tree	LF, PSF	LC	NE
Pentaphylacaceae	*Adinandra sarosanthera* Miq.	Tetiup, Kelat Pamah, Petuta Bukit, Pongpong Raya, Samak	Tree	LF	NE	NE
	*Ternstroemia wallichiana* (Griff.) Engl.	Medang Bunga Lawang	Tree	LF	VU	NE
Phyllanthaceae	*Antidesma cuspidatum* Müll.Arg.	Beruni, Berunai, Sebasah Bukit	Tree	LF, FSF	NE	NE
	*Antidesma ghaesembilla* Gaertn.	Beruni, Balong Ayam, Guncak	Tree	LF, PSF	LC	NE
	*Breynia racemosa* (Blume) Müll.Arg.	Hujan panas, Ambin Kera, Peringat, Saga, Sumbar	Tree	LF, BV	LC	NE
	*Glochidion littorale* Blume	Jambu Kera	Tree	BV, MSF	LC	NE
Pittosporaceae	*Pittosporum ferrugineum* W.T.Aiton.	Belalang Puak, Cemperai Ikan, Chabek Hantu	Tree	BV	LC	LC
Primulaceae	*Aegiceras corniculatum* (L.) Blanco	Kuku Lang, Kacang-kacang, Teruntun	Tree	MSF	LC	NE
	*Ardisia crenata* Sims.	Mata ayam, Akar Bebuluh, Mata Pelandok, Sirih Puyuh	Shrub		NE	NE
	*Ardisia elliptica* Thunb.	Mata pelanduk, Buah Letus, Daun Bisa Hati, Jambulan Pantai, Jangkang, Kayu Lampilan, Mempenai	Tree	FSF	NE	E
	*Embelia ribes* Burm.f.	-	Shrub/Climbing shrub	LF	NE	NE
	*Rapanea porteriana* (Wall. ex A.DC.) Mez.	Kicar, Dedahruang	Tree	MSF	NE	NE
Rhizophoraceae	*Bruguiera cylindrica* (L.) Blume	Berus-berus, Bakau Putih, Berus Putih	Tree	MSF	LC	NE
	*Bruguiera gymnorhiza* (L.) Lam. ex Savigny	Tumu Merah, Lenggadai	Tree	MSF	LC	NE
	*Bruguiera sexangula* (Lour.) Poir	Tumu Putih, Tumu Berau, Mata Buaya, Putut	Tree	MSF	LC	NE
	*Bruguiera hainesii* C.G.Rogers	Berus Mata Buaya	Tree	MSF	CR	NE
	*Bruguiera* x *rhynchopetala* (W.C.Ko) N.C.Duke & X.J.Ge	Tumu Hibrid	Tree	MSF	DD	NE
	*Carallia brachiata* (Lour.) Merr.	Sisik Puyu, Merpuing, Meransi	Tree	LF, MSF, BV	NE	NE
Rhizophoraceae	*Ceriops tagal* (Pers.) C.B.Rob.	Tengar, Tengar Samak	Tree	MSF	LC	NE
	*Ceriops zippeliana* Blume	Tengar	Tree	MSF	LC	NE
	*Gynotroches axillaris* Blume	Mata Keli, Bulu Bulu, Kandis Batu	Tree		NE	NE
	*Rhizophora apiculata* Blume	Bakau Minyak, Bakau Akik, Bakau Tandok, Bangkita	Tree	MSF	LC	NE
	*Rhizophora mucronata* Lam.	Bakau Kurap, Bakau Belukap, Bakau Gelukap, Bakau Jankar	Tree	MSF	LC	NE
	*Rhizophora* x *annamalayana* Kathir.	Bakau Hibrid	Tree	MSF	DD	NE
Rhamnaceae	*Colubrina asiatica* (L.) Brongn.	Bidara Laut , Peria Pantai	Shrub	BV	NE	NE
Rubiaceae	*Canthium confertum* Korth.	Kemuning Jantan	Tree	BV,LF	NE	NE
	*Catunaregam spinosa* (Thunb.) Tirveng.	Duri Timbang Tahil	Tree	CDF	NE	NE
	*Catunaregam tomentosa* (Blume ex DC.) Tirveng.	Duri Timbang Tahil	Tree	BV	NE	NE
	*Gardenia tubifera* Wall. ex Roxb.	Mentiong Paya, Chempaka Hutan, Delima Hutan	Tree	LF	DD	NE
	*Guettarda speciosa* L.	Selar Malam, Bebaru Laut, Katapang Pasir	Tree	BV	NE	NE
	*Gynochthodes sublanceolata* Miq.	Akar sulong, Akar Lampai Hitam	Shrub	LF	NE	NE
	*Oldenlandia herbacea* (L.) Roxb.	Siku-siku	Terrestrial herb	BV	DD	NE
	*Hydnophytum formicarum* Jack	Kepala Berok, Sarang Semut	Epiphytic shrub	PSF, BV	NE	NE
	*Hypobathrum racemosum* (Roxb.) Kurz.	Empawang Putih	Tree	LF	NE	NE
	*Ixora concinna* R.Br.ex Hook.f.	Jenjarum	Shrub	LF	NE	NE
	*Ixora grandifolia* Zoll. & Moritzi	Jenjarum, Jarum Hutan	Shrub	LF	DD	NE
	*Kailarsenia tentaculata* (Hook.f.) Tirveng.	Kecubong Paya, Kepayang Air	Shrub	RV	NE	NE
	*Morinda citrifolia* L.	Mengkudu Daun Kecil, Noni, Mengkudu, Mengkudu Besar	Tree	DV	NE	NE
	*Morinda umbellata* L.	Mengkudu akar, Mengkudu Hutan	Climbing shrub	BV,LF	NE	NE
	*Ixora congesta* Roxb.	Pecah Periok, Bunga Penaga Riam, Jarum Saluang	Shrub	LF	NE	NE
Rubiaceae	Ixora umbellata var. multibracteata (H.Pearson ex King & Gamble) Corner	Pecah Periok	Shrub	LF	DD	NE
	*Mussaenda glabra* Vahl	Balik Adap	Shrub	FSF, DV	NE	NE
	*Myrmecodia tuberosa* Jack	Periok Hantu	Shrub	MSF, FSF	NE	NE
	*Oxyceros longiflorus* (Lam.) T.Yamaz.	Akar Kekait, Akar Bedara Laut, Akar Duri	Climbing shrub	MSF	DD	NE
	*Psychotria sarmentosa* Blume	Akar Daldaru, Kaum Kopi	Climbing shrub	LF, FSF	NE	NE
	*Scyphiphora hydrophyllacea* C.F.Gaertn.	Chengam	Shrub	MSF	LC	NE
	*Tarenna fragrans* (Blume) Koord. & Valeton	Julong-julong Jantan	Tree	LF,RV	DD	NE
	*Timonius flavescens* (Jacq.) Baker	Kurau, Kaum Kopi	Tree	LF,PSF	LC	NE
	*Uncaria acida* (W.Hunter) Roxb.	Gambir-gambir	Climbing shrub	LF	NE	NE
Rutaceae	*Acronychia pedunculata* (L.) Miq.	Jenjagong	Tree	LF	LC	NE
	*Melicope lunu-ankenda* (Gaertn.) T.G.Hartley	Tenggek burung, Pepauh, Chabang Tiga	Shrub	LF	LC	NE
Salicaceae	*Flacourtia rukam* Zoll. & Moritzi	Rukam	Tree	RV	NE	NE
	*Scolopia macrophyll*a (W.& A.) Clos	Rukam Hutan	Tree	MSF, RV	NE	NE
Santalaceae	*Dendrotrophe buxifolia* (Blume) Miq.	Setong Jundor	Parasitic shrub	BV	DD	NE
	*Viscum orientale* Willd.	Dedalu	Parasitic shrub	MSF	DD	NE
	*Viscum ovalifolium* DC.	Dedalu Emping, Api-api	Parasitic shrub	MSF	LC	LC
Sapotaceae	*Palaquium obovatum* (Griff.) Engl.	Taban Putih, Nyatoh, Nyatoh Putih	Tree	LF, FSF	LC	NE
	*Planchonella obovata* (R.Br.) Pierre	Nenasi, Misi, Nyatoh Laut, Nyatoh Kuning	Tree	MSF, BV	NE	NE
Sapindaceae	*Allophylus cobbe* (L.) Raeusch	Buah Penancang, Congkol, Cungkil, Kasai, Kasai Daun Kecil	Shrub	LF, MSF, BV	NE	NE
	*Dodonaea viscosa* (L.) Jacq.	Serengan laut, Kayu Bertih	Tree	RV, BV	LC	NE
	*Guioa bijuga* (Hiern) Radlk.	Senyamok	Tree	LF	NE	NE
	*Guioa pleuropteris* (Blume) Radlk.	Senyamok, Samak, Kelentit, Nyamuk Laut, Pena-pena, Sempayan Ular	Tree	LF, RV	NE	NE
Sapindaceae	*Lepisanthes rubiginosa* (Roxb.) Leenh.	Mertajam, Kelat Layu, Terajah	Tree	RV	LC	NE
	*Mischocarpus sundaicus* Blume	Suji	Tree	LF	NE	NE
Simaroubaceae	*Eurycoma longifolia* Jack	Tongkat Ali, Bidara Merah, Lempedu Pahit, Pasak Bumi, Setunjang Bumi	Tree	LF, BV	NE	NE
	*Quassia indica* (Gaertn.) Noot.	Kayu Pahit, Gatip pahit, Kacang-kacang	Tree	PSF, MSF	NE	NE
Symplocaceae	*Symplocos adenophylla* Wall. ex G.Don	Semugum, Jiak	Tree	LF	DD	NE
Thymelaeaceae	*Wikstroemia indica* (L.) C.A.Mey	Depu	Shrub	DV	NE	NE
Vitaceae	*Cayratia trifolia* (L.) Domin	Galing-galing, Lakum	Shrub	LF, DV	NE	NE
	*Cissus hastata* Miq.	Akar Asam Riang, Akar Kerayong	Shrub	LF	NE	NE
Ximeniaceae	*Ximenia americana* L.	Bedara laut	Tree	BV	LC	NE
**MONOCOTYLEDONS**						
Aizoaceae	*Sesuvium portulacastrum* (L.) L.	Gelang Laut, Gelang Pasir, Saruni Air	Terrestrial herb	MSF, CDF	NE	LC
Amaryllidaceae	*Crinum asiaticum* L.	Bakong, Tembaga Suasa	Terrestrial herb	RV, LF, FSF	NE	NE
Araceae	*Cryptocoryne ciliata* (Roxb.) Schott.	Keladi Payau	Aquatic herb	MSF	LC	NE
	*Cryptocoryne cordata* Griff.	-	Aquatic herb	RV	LC	NE
	*Cryptocoryne griffithii* Schott.	-	Aquatic herb	PSF	NE	NE
	*Lasia spinosa* (L.) Thw.	Geli-geli	Terrestrial herb	PSF	LC	NE
	*Scindapsus hederaceus* (Zoll. & Moritizi) Miq.	Akar Lebang Aleh	Climbing herb	PSF	NE	NE
Arecaceae	*Calamus erinaceus* (Becc.) J.Dransf.	Rotan Bakau	Palm	MSF	NE	NE
	*Licuala spinosa* Wurmb.	Palas, Palas Duri	Palm	MSF	NE	NE
	*Nypa fruticans* Wurmb.	Nipah	Palm	MSF	LC	NE
	*Oncosperma tigillarium* (Jack) Ridl.	Nibong, Ibas, Linau, Nibung, Nibong	Palm	MSF	NE	NE
	*Phoenix paludosa* Roxb.	Kedangsa, Dangsa	Palm	MSF	NT	NE
Asparagaceae	*Dracaena porteri* Baker	Jarum-jarum	Shrub	LF, FSF	NE	NE
Commelinaceae	*Cyanotis cristata* D.Don	Petungan	Terrestrial herb	BV	LC	NE
Cymodoceaceae	*Halodule pinifolia* (Miki) Hartog	-	Aquatic herb	FSF	LC	NE
Cyperaceae	*Bulbostylis barbata* (Rottb.) C.B.Clarke	Rumput rusiga	Terrestrial herb	FSF	NE	NE
	*Cyperus distans* L.f.	Rumput rusiga	Aquatic herb	RV,PSF	LC	NE
	*Cyperus digitatus* Roxb.	Rumput rusiga	Aquatic herb	PSF	LC	NE
	*Cyperus javanicus* Houtt.	Rumput rusiga	Terrestrial herb	MSF	NE	NE
	*Cyperus rotundus* L.	Rumput rusiga	Terrestrial herb	RV,BV	LC	NE
	*Cyperus stoloniferus* Retz.	Rumput rusiga	Terrestrial herb	BV	LC	NE
	*Eleocharis geniculata* (L.) Roem. & Schult.	Rumput rusiga	Terrestrial herb	RV	LC	NE
	*Eleocharis ochrostachys* Steud.	Rumput rusiga	Terrestrial herb	PSF	LC	NE
	*Eleocharis retroflexa* (Poir.) Urb.	Rumput rusiga	Terrestrial herb	RV	LC	NE
	*Fimbristylis acuminata* Vahl	Rumput rusiga	Terrestrial herb	RV	LC	NE
	*Fimbristylis cymosa* R.Br.	Rumput rusiga	Terrestrial herb	MSF	LC	NE
	*Fimbristylis pauciflora* R.Br.	Rumput rusiga	Terrestrial herb	LF	NE	NE
	*Fuirena umbellata* Rottb.	Rumput rusiga	Terrestrial herb	RV	LC	NE
	*Lepironia articulata* (Retz.) Domin	Kercut/Kerchut	Aquatic herb	FSF	NE	NE
	*Rhynchospora brownii* Roem. & Schult.	Rumput rusiga	Terrestrial herb	HV, BRIS	NE	NE
	*Remirea maritima* Aubl.	Rumput rusiga	Terrestrial herb	CDF,BV	NE	NE
	*Scleria levis* Retz.	Rumput rusiga	Terrestrial herb	FSF,DV	NE	NE
	*Scleria poaeformis* Retz.	Rumput rusiga	Terrestrial herb	FSH,DV	NE	NE
Eriocaulaceae	*Eriocaulon truncatum* Buch.-Ham. ex Mart.	-	Aquatic herb	DV	LC	NE
	*Eriocaulon willdenovianum* Moldenke	-	Aquatic herb	DV	NE	NE
Flagellariaceae	*Flagellaria indica* L.	Rotan Dini, Rotan Tikus, Rotan Kera	Climbing shrub	MSF, FSF	NE	NE
Hanguanaceae	*Hanguana malayana* (Jack) Merr.	Bakong	Aquatic herb	RV, FSF	LC	NE
Hydrocharitaceae	*Blyxa aubertii* Rich.	-	Aquatic herb	FSF	LC	NE
	*Halophila beccarii* Aschers.	-	Aquatic herb	MSF	VU	NE
	*Halophila minor* (Zollinger) den Hartog	-	Aquatic herb	BV	LC	NE
	*Halophila ovalis* (R.Brown) J.D.Hooker	-	Aquatic herb	BV	LC	NE
Orchidaceae	*Acriopsis liliifolia* (J. Koenig) Ormerod	Orkid	Epiphytic herb	BRIS	NE	NE
	*Appendicula cornuta* Blume	Orkid	Epiphytic herb	BRIS	NE	NE
	*Appendicula uncata* Ridl.	Orkid	Epiphytic herb	BRIS	NE	NE
	*Ania penangiana* (Hook.f.) Summerh.	Orkid	Terrestrial herb	BRIS	NE	NE
	*Arachnis flos-aeris* (L.) Rchb.f.	Orkid	Epiphytic herb	BRIS	NE	NE
	*Arachnis hookeriana* (Rchb.f.) Rchb.f.	Orkid	Epiphytic herb	BRIS	NE	NE
	*Bromheadia finlaysoniana* (Lindl.) Miq	Orkid	Terrestrial herb	BRIS	LC	NE
	*Bulbophyllum acuminatum* (Ridl.) Ridl.	Orkid	Epiphytic herb	BRIS	NE	NE
	*Bulbophyllum apodum* Hook.f.	Orkid	Epiphytic herb	BRIS	NE	NE
	*Bulbophyllum clandestinum* Lindl.	Orkid	Epiphytic herb	BRIS	NE	NE
	*Bulbophyllum fenestratum* J.J.Sim	Orkid	Epiphytic herb	BRIS	NE	NE
	*Bulbophyllum macranthum* Lindl.	Orkid	Epiphytic herb	BRIS	LC	NE
	*Bulbophyllum patens* King ex Hook.f.	Orkid	Epiphytic herb	BRIS	NE	NE
	*Bulbophyllum planibulbe* (Ridl.) Ridl.	Orkid	Epiphytic herb	BRIS	NE	NE
	*Bulbophyllum purpurascens* Teijsm. & Binn.	Orkid	Epiphytic herb	BRIS	NE	NE
	*Bulbophyllum trigonopus* (Rchd.f) P.T.Ong	Orkid	Epiphytic herb	BRIS	NE	NE
	*Bulbophyllum vaginatum* (Lindl.) Rchb.f.	Orkid	Epiphytic herb	BRIS	NE	NE
	*Callostylis pulchella* (Lindl.) S.C.Chen & Z.H.Tsi	Orkid	Epiphytic herb	HV, BRIS	NE	NE
	*Ceratostylis subulata* Blume	Orkid	Epiphytic herb	BRIS	LC	NE
	*Claderia viridiflora* Hook.f.	Orkid	Terrestrial herb	BRIS	NE	NE
	*Cleisostoma teretifolium* Teijsm. & Binn.	Orkid	Epiphytic herb	BRIS	NE	NE
	*Coelogyne foerstermannii* Rchb.f.	Orkid	Epiphytic herb	HV, BRIS	LC	NE
	*Cymbidium finlaysonianum* Lindl.	Orkid	Epiphytic herb	HV, BRIS	NE	NE
	*Cymbidium rectum* Ridl.	Orkid	Epiphytic herb	HV, BRIS	NE	NE
	*Dendrobium acerosum* Lindl.	Orkid	Epiphytic herb	BRIS	NE	NE
	*Dendrobium aloifolium* (Blume) Rchb.f.	Orkid	Epiphytic herb	BRIS	LC	NE
Orchidaceae	*Dendrobium angustifolium* (Blume) Lindl.	Orkid	Epiphytic herb	BRIS	NE	NE
	*Dendrobium clavator* Ridl.	Orkid Merpati	Epiphytic herb	MSF, BRIS	NE	NE
	*Dendrobium crumenatum* Sw.	Orkid	Epiphytic herb	BRIS	NE	NE
	*Dendrobium lamellatum* (Blume) Lindl.	Orkid	Epiphytic herb	BRIS	NE	NE
	*Dendrobium leonis* (Lindl.) Rchb.f.	Orkid	Epiphytic herb	BRIS	NE	NE
	*Dendrobium pachyphyllum* (Kuntze) Bakh.f.	Orkid	Epiphytic herb	BRIS	NE	NE
	*Dendrobium rhodostele* Ridl.	Orkid	Epiphytic herb	BRIS	NE	NE
	*Dendrobium secundum* (Blume) Lindl. ex Wall.	Orkid	Epiphytic herb	BRIS	NE	NE
	*Dendrolirium lasiopetalum* (Willd.) S.C.Chen & J.J.Wood	Orkid	Epiphytic herb	BRIS	NE	NE
	*Eulophia graminea* Lindl.	Orkid	Terrestrial herb	BRIS	NE	NE
	*Grammatophyllum speciosum* Blume	Orkid Harimau	Epiphytic/Terrestrial herb	BRIS	NE	NE
	*Liparis ferruginea* Lindl.	Orkid	Terrestrial herb	FWF	NE	NE
	*Luisia jonesii* J.J.Sm.	Orkid	Epiphytic herb	BRIS	NE	NE
	*Oberonia padangensis* Schltr.	Orkid	Epiphytic herb	BRIS	NE	NE
	*Papilionanthe hookeriana* (Rchb.f.) Schltr.	Orkid	Epiphytic herb	FWF	NE	NE
	*Phalaenopsis pulcherrima* (Lindl.) J.J.Sm.	Orkid	Terrestrial herb	HV, BRIS	NE	NE
	*Pinalia atrovinosa* (Carr) Schuit., Y.P.Ng & H.A.Pedersen	Orkid	Epiphytic herb	BRIS	NE	NE
	*Pinalia floribunda* (Lindl.) Kuntze	Orkid	Epiphytic herb	BRIS	NE	NE
	*Pinalia tenuiflora* (Ridl.) J.J.Wood	Orkid	Epiphytic herb	BRIS	NE	NE
	*Plocoglottis lowii* Rchb.f.	Orkid	Terrestrial herb	BRIS	NE	NE
	*Polystachya concreta* (Jacq.) Garay & H.R.Sweet	Orkid	Epiphytic herb	BRIS	NE	NE
	*Renanthera elongata* (Blume) Lindl.	Orkid	Epiphytic herb	BRIS	NE	NE
	*Strongyleria pannea* (Lindl.) Schuit., Y.P.Ng & H.A.Pedersen	Orkid	Terrestrial herb	BRIS	NE	NE
	*Taeniophyllum pusillum* (Willd.) Seidenf. & Ormerod	Orkid hantu	Epiphytic herb	MSF,BRIS	NE	NE
Orchidaceae	*Thrixspermum amplexicaule* (Blume) Rchb.f.	Orkid	Epiphytic herb	FSF	NE	NE
	*Thrixspermum calceolus* (Lindl.) Rchb.f.	Orkid	Epiphytic herb	BRIS	NE	NE
	*Thrixspermum centipeda* Lour.	Orkid	Epiphytic herb	BRIS	NE	NE
	*Thrixspermum scopa* (Rchb.f. ex Hook.f.) Holttum	Orkid	Epiphytic herb	BRIS	NE	NE
	*Thrixspermum trichoglottis* (Hook.f.) Kuntze	Orkid	Epiphytic herb	BRIS	NE	NE
	*Vanilla griffithii* Rchb.f.	Telinga Kerbau	Terrestrial/Epiphytic herb	BRIS	NE	NE
Pandanaceae	*Pandanus atrocarpus* Griff.	Mengkuang Paya	Palm-like	LF	NE	NE
	*Pandanus helicopus* Kurz ex Miq.	Mengkuang Paya, Rasau	Palm-like	LF	NE	NE
	*Pandanus tectorius* Parkinson	Mengkuang Laut, Pandan Duri, Pandan Laut	Palm-like	MSF	LC	NE
	*Pandanus yvanii* Solms.	Mengkuang Paya	Palm-like	LF	NE	NE
Philydraceae	*Philydrum lanuginosum* Banks & Sol. ex Gaertn.	Rumput Kipas	Terrestrial herb	FWS	NE	NE
Poaceae	*Chloris barbata* Sw.	Rumput Jari Kembong	Terrestrial herb	CDF,BV	NE	NE
	*Chrysopogon aciculatus* (Retz.) Trin.	Kemuncup	Terrestrial herb	CDF,BV	NE	NE
	*Chrysopogon serrulatus* Trin.	Kemuncup Besar	Terrestrial herb	CDF, BV	NE	NE
	*Cynodon dactylon* (L.) Pers.	Rumput Minyak	Terrestrial herb	BV, DV	NE	NE
	*Eleusine indica* (L.) Gaertn.	Sambau	Terrestrial herb	DV	LC	NE
	*Eriachne pallescens* R.Br.	-	Terrestrial herb	BV, DV	NE	NE
	*Imperata cylindrica* (L.) P.Beauv.	Lalang	Terrestrial herb	BV	LC	NE
	*Ischaemum muticum* L.	Rumput Tembaga Jantan, Rumput Terutus Tembaga	Terrestrial herb	BV	LC	NE
	*Leersia hexandra* Sw.	-	Terrestrial herb	FSF	LC	NE
	*Paspalum orbiculare* G.Forst.	-	Terrestrial herb	FSF	NE	NE
	*Sacciolepis indica* (L.) Chase	-	Terrestrial herb	CDF,FSF	DD	NE
	*Zoysia matrella* (L.) Merr.	-	Terrestrial herb	BV	NE	NE
Restionaceae	*Dapsilanthus disjunctus* (Mast.) B.G.Briggs – & L.A.S.Johnson	Terrestrial herb	BV	NE	NE	
Xyridaceae	*Xyris complanata* R.Br.	-	Terrestrial herb	RV	LC	NE
	*Xyris pauciflora* Willd.	-	Terrestrial herb	RV	LC	NE
Zingiberaceae	*Alpinia aquatica* (Retz.) Roscoe	Munkanang	Terrestrial herb	FSF, RV	NE	NE
	*Alpinia conchigera* Griff.	Lengkuas Ranting, Lengkuas Geting, Lengkuas Kecil, Lengkuas Padi	Terrestrial herb	FSF, DV	NE	NE
	*Alpinia galanga* (L.) Willd.	Lengkuas	Terrestrial herb	LF	NE	NE
	*Alpinia javanica* Blume	Lengkuas Hutan	Terrestrial herb	LF	LC	NE
	*Alpinia oxymitra* K.Schum.	-	Terrestrial herb	LF	NE	NE
